# High-Frequency Stimulation of the Rat Entopeduncular Nucleus Does Not Provide Functional or Morphological Neuroprotection from 6-Hydroxydopamine

**DOI:** 10.1371/journal.pone.0133957

**Published:** 2015-07-29

**Authors:** D. Luke Fischer, Timothy J. Collier, Allyson Cole-Strauss, Susan L. Wohlgenant, Jack W. Lipton, Kathy Steece-Collier, Fredric P. Manfredsson, Christopher J. Kemp, Caryl E. Sortwell

**Affiliations:** 1 Department of Translational Science and Molecular Medicine, College of Human Medicine, Michigan State University, Grand Rapids, MI, United States of America; 2 MD/PhD and Neuroscience Graduate Programs, College of Human Medicine, Michigan State University, Grand Rapids, MI, United States of America; Georgia Institute of Technology, UNITED STATES

## Abstract

Deep brain stimulation (DBS) is the most common neurosurgical treatment for Parkinson’s disease (PD). Whereas the globus pallidus interna (GPi) has been less commonly targeted than the subthalamic nucleus (STN), a recent clinical trial suggests that GPi DBS may provide better outcomes for patients with psychiatric comorbidities. Several laboratories have demonstrated that DBS of the STN provides neuroprotection of substantia nigra pars compacta (SNpc) dopamine neurons in preclinical neurotoxin models of PD and increases brain-derived neurotrophic factor (BDNF). However, whether DBS of the entopeduncular nucleus (EP), the homologous structure to the GPi in the rat, has similar neuroprotective potential in preclinical models has not been investigated. We investigated the impact of EP DBS on forelimb use asymmetry and SNpc degeneration induced by 6-hydroxydopamine (6-OHDA) and on BDNF levels. EP DBS in male rats received unilateral, intrastriatal 6-OHDA and ACTIVE or INACTIVE stimulation continuously for two weeks. Outcome measures included quantification of contralateral forelimb use, stereological assessment of SNpc neurons and BDNF levels. EP DBS 1) did not ameliorate forelimb impairments induced by 6-OHDA, 2) did not provide neuroprotection for SNpc neurons and 3) did not significantly increase BDNF levels in any of the structures examined. These results are in sharp contrast to the functional improvement, neuroprotection and BDNF-enhancing effects of STN DBS under identical experimental parameters in the rat. The lack of functional response to EP DBS suggests that stimulation of the rat EP may not represent an accurate model of clinical GPi stimulation.

## Introduction

Parkinson’s disease (PD) affects nearly one percent of the population over the age of sixty-five [1]. The most common symptoms are bradykinesia, postural instability, rigidity and resting tremor with motor dysfunction being the primary cause for diagnosis, even though a patient may also have depression, cognitive dysfunction, anosmia or other symptoms at clinical presentation [2]. These motor symptoms primarily are a result of degeneration of the dopaminergic cells of the substantia nigra pars compacta (SNpc) and their projections to the striatum. As a result, the current mainstay pharmacotherapy of levodopa (L-DOPA) attempts to bolster nigrostriatal dopaminergic transmission. However, as disease progression continues, dopaminergic pharmacotherapy has decreased symptomatic efficacy and can yield troubling, involuntary movement known as dyskinesia [3], making the identification of neuroprotective therapies critical. Beyond pharmacotherapy, the surgical approach of deep brain stimulation (DBS) of the subthalamic nucleus (STN) is used with increasing frequency as a way to manage many PD motor symptoms.

Since the advent of DBS, neurosurgeons have often chosen to target the STN for both surgical and symptomatic goals. STN DBS also results in a reduction of the needed L-DOPA dosage, thereby lessening the severity of drug-induced dyskinesia [4–6]. Whereas traditionally the STN has been the preferred implantation site, similar success in treating the motor symptoms of PD with DBS targeting the globus pallidus interna (GPi) has been reported ([5], see also [6–8]). In some instances STN DBS has been associated with depressive symptoms or executive dysfunction post-surgery [9]. The potential for DBS targeted to the STN to exacerbate the existing comorbidities of depression or cognitive dysfunction has led to new consideration of DBS targets based on patient-specific motor and non-motor symptoms [4].

Despite the symptomatic efficacy of DBS, our understanding of its impact on ongoing nigral degeneration remains limited. This is in part due to the practice of using DBS as a last-resort treatment in late-stage PD. Patients who elect surgery receive DBS on average fourteen years after diagnosis [6]. In 2013 the results from a randomized clinical trial in PD patients with mid-stage PD (7.5 years) favored STN DBS over optimized medical therapy [10]. This study will likely shift clinical practice to offer DBS to *mid-stage* PD patients. Yet 50–60% of nigral dopamine (DA) neurons have degenerated seven years post PD diagnosis [11]. Studies in which STN DBS is applied in early-stage PD will be required to investigate its neuroprotective potential. STN DBS was recently shown to be efficacious and safe in early-stage PD [12–16]. The increased focus on early DBS illustrates the significance of preclinical studies aimed at investigating this phenomenon. Further, given the difficulties with assessing neuroprotection in the clinic, preclinical studies can lead the way in the development and assessment of potentially disease-modifying therapies [17, 18].

Previous work in a rat model of long-term STN DBS [19] has yielded three distinct findings. First, STN DBS is associated with significant improvements in contralateral forelimb deficits induced by intrastriatal 6-hydroxydopamine (6-OHDA) injections, an animal model of PD in which DA neurodegeneration is induced by oxidative stress [20]. Second, STN DBS halts ongoing nigral DA neuron degeneration induced by intrastriatal 6-OHDA. Of importance, the degeneration is halted at the stimulation start time (two weeks post lesion) and midway through the course of expected degeneration. Third, STN DBS significantly increased brain-derived neurotrophic factor (BDNF) in the nigrostriatal system, the primary motor cortex (M1) and the entopeduncular nucleus (EP), suggesting its involvement in symptom amelioration or neuroprotection [21]. These preclinical results demonstrate that long-term STN DBS has the potential to impact plasticity within basal ganglia circuitry or may provide neuroprotection against further nigral loss. However, whether DBS targeted to the EP (the homologous structure to the primate GPi in the rat) will similarly provide functional improvements in forelimb use, facilitate nigral DA neuroprotection or alter BDNF levels is unknown. In the present study we examined the impact of long-term EP DBS on the progression of 6-OHDA-induced nigral degeneration and deficits in contralateral forelimb use.

## Methods

### Animals

A total of thirty-two, male, Sprague-Dawley rats (Harlan, 200–250 g) were used in these studies. Rats were only included in the final analysis if they successfully completed the two-week stimulation interval and exhibited proper electrode placement in the EP. Thirteen rats received intrastriatal 6-hydroxydopamine (6-OHDA) and EP stimulators; nineteen rats were unlesioned with EP stimulators. Animals were allowed food and water *ad libitum* and housed in reverse dark-light cycle conditions in an AAALAC approved facility. This study was specifically approved by the Institutional Animal Care and Use Committee of Michigan State University.

### Experimental overview

**Fig 1:**
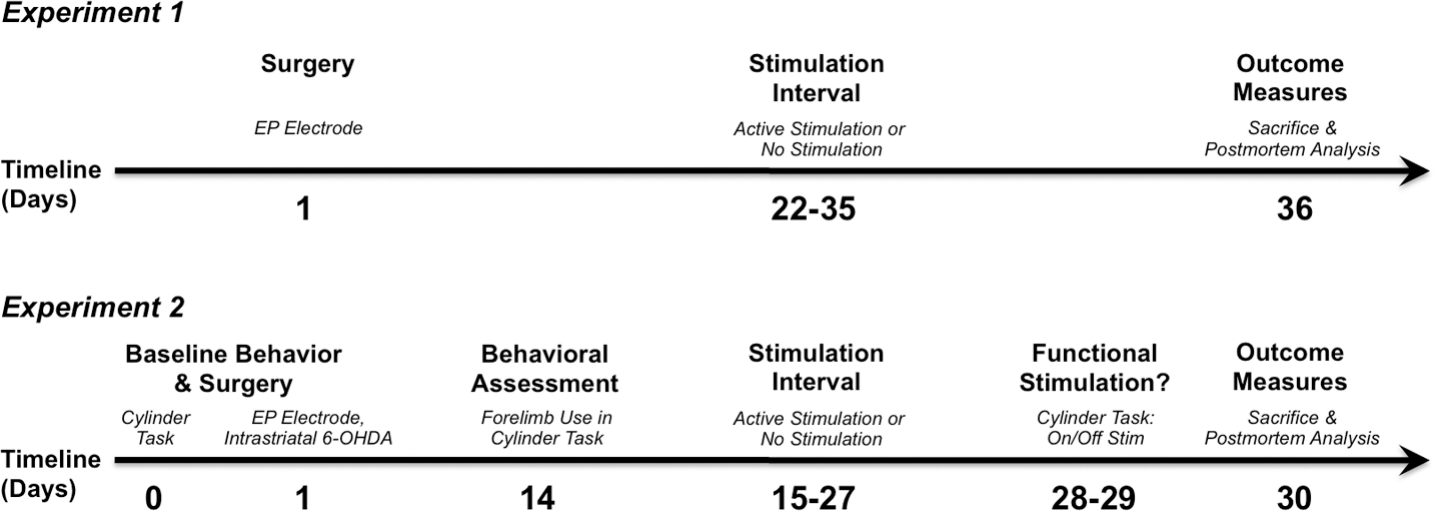
Experimental overview for EP DBS. *Experiment 1*. On Day 1, rats received an electrode implanted in the EP. After three weeks of recovery, rats were randomly assigned to ACTIVE or INACTIVE stimulation for a two-week interval. Rats tolerated stimulation of the EP for two weeks as they otherwise would for STN DBS for the same duration. Rats were sacrificed and perfused on Day 36. *Experiment 2*. On Day 0, rats were assessed for baseline forelimb asymmetry using the cylinder task. On Day 1, rats received unilateral, intrastriatal 6-OHDA and an electrode was implanted during the same surgical session in the EP ipsilateral to the lesion. After two weeks of nigrostriatal degeneration (≈50% loss of SNpc neurons, as determined in [20]), rats were reassessed for forelimb asymmetry, and rats with sufficient deficits in contralateral paw use were randomly assigned to receive ACTIVE or INACTIVE stimulation for a two-week interval. On Day 28, rats were reassessed using the cylinder task (“Stim On” condition), and after a twenty-four-hour washout after the cessation of stimulation, the rats were again assessed using the cylinder task (“Stim Off” condition). Rats were sacrificed and perfused on Day 30.

The following experiments sought to replicate previously published work [20, 21] except that the EP was targeted rather than the STN. Care was taken to conduct the experiments as closely as possible to the original work to allow for comparisons. The overall experimental design is depicted in Fig 1.

### Experiment 1: stimulation of the EP in unlesioned rats

All rats were implanted unilaterally with electrodes to the EP and allowed a recovery period of three weeks. Rats were then randomly divided into ACTIVE and INACTIVE stimulation groups. Rats assigned to the ACTIVE group were connected to an external stimulation source and received continuous stimulation to the EP for two weeks; the INACTIVE group did not receive stimulation during the same interval. After the two-week period of stimulation, rats were sacrificed within six hours of cessation of stimulation. Brain structures including the M1 cortex (8 ACTIVES vs. 10 INACTIVES), striatum (8 ACTIVES vs. 9 INACTIVES), hippocampus (3 ACTIVES vs. 7 INACTIVES), thalamus (4 ACTIVES vs. 7 INACTIVES) and substantia nigra (8 ACTIVES vs. 10 INACTIVES) were microdissected and processed for enzyme-linked immunosorbent assay (ELISA) for BDNF.

### Experiment 2: stimulation of the EP following intrastriatal 6-OHDA

At least twenty-four hours prior to surgery, forelimb use in the cylinder task was assessed. Rats then received unilateral, intrastriatal injections of 6-OHDA and ipsilateral implantation of an electrode to the EP during the same surgical session. After a two-week recovery period, all rats were reassessed in the cylinder task to determine lesion status, and those that were functionally lesioned (i.e., a minimum 20% reduction in contralateral forelimb use compared to baseline) were randomly divided into ACTIVE and INACTIVE stimulation groups. Rats assigned to the ACTIVE group were connected to an external stimulation source and received continuous stimulation to the EP for two weeks; the INACTIVE group did not receive stimulation during the same interval. At the end of the two-week period of stimulation, rats were assessed using the cylinder task with stimulation and twenty-four hours after cessation of stimulation (3 ACTIVES vs. 7 INACTIVES). All rats were sacrificed within forty-eight hours after cessation of stimulation (4 ACTIVES vs. 7 INACTIVES for morphological analysis).

### Intrastriatal 6-OHDA injections

Intrastriatal 6-OHDA injections were conducted as described previously [20]. Specifically, rats were anesthetized prior to surgery with Equithesin (0.3 ml / 100 g body weight i.p.; chloral hydrate 42.5 mg/ml + sodium pentobarbital 9.72 mg/ml), and then they received two unilateral, intrastriatial injections (AP +1.6 mm, ML +2.4 mm, DV −4.2 mm and AP +0.2 mm, ML +2.6 mm, DV −7.0 mm relative to bregma) of 6-OHDA (MP Biomedicals, Solon, OH; 5.0 μg/μl 6-OHDA in 0.02% ascorbic acid, 0.9% saline solution, injection rate 0.5 μl/minute, 2.0 μl per site). Drill holes were filled with gel foam or bone wax to prevent entry of cement from electrode placement. These 6-OHDA lesion parameters result in ≈50% loss of substantia nigra pars compacta (SNpc) tyrosine hydroxylase immunoreactive (THir) neurons after two weeks that progresses to ≈75% loss after four weeks [20].

### Electrode implantation

In ‘Experiment 1’ naïve rats were implanted with electrodes, whereas in ‘Experiment 2’ rats were implanted with electrodes immediately following intrastriatal 6-OHDA injections. Specifically, rats were unilaterally implanted (ipsilateral to 6-OHDA injections) with a bipolar, concentric microelectrode (inner electrode projection 1.0 mm, inner insulated electrode diameter 0.15 mm, outer electrode gauge 26, Plastics One, Roanoke, VA) targeted to the dorsal border of the EP (AP −2.4 mm, ML +3.0 mm, relative to bregma and DV −6.6 mm, relative to the dura mater). Burr holes were drilled in the skull, and the electrode was fixed in place using bone screws and dental acrylic. Electrodes were lowered to coordinates corresponding to the dorsal border of the EP in order to minimize damage to the nucleus.

### Behavioral testing

Spontaneous forelimb use was assessed using the cylinder task as previously described [21–23] prior to surgery, two weeks following surgery and four weeks following surgery both on and off stimulation. Other behavioral measures were not employed due to their incompatibility with the external hardware required for continuous, long-term stimulation. Briefly, during the dark cycle rats were videotaped and placed in a clear plexiglass cylinder until twenty, weight-bearing forelimb placements on the side of the cylinder occurred, or until a maximum trial time of five minutes had elapsed. To determine if forelimb preference was present, the number of contralateral, ipsilateral, and simultaneous paw placements was quantified. Data are reported as the percentage of contralateral (to 6-OHDA and electrode) forelimb use: [(contralateral + ½ both)/(ipsilateral + contralateral + both)] x 100%. Rats with a unilateral nigrostriatal lesion will show a bias toward using the ipsilateral limb. Extent of lesion was evaluated two weeks post surgery, and a forelimb deficit was defined as possessing a minimum of a one-fifth reduction in contralateral forepaw use compared to baseline (i.e., 50% contralateral forelimb use at baseline would meet the lesion threshold if reduced to 40%).

### Long-term stimulation

Rats in the ACTIVE group received continuously delivered stimulation in a freely moving setup as previously described [20]. Stimulation was generated by an Accupulser Signal Generator (World Precision Instruments, Sarasota, FL) via a battery-powered Constant Current Bipolar Stimulus Isolator (World Precision Instruments, Sarasota, FL). Stimulation parameters consisted of a frequency of 130 Hz, a pulse width of 60 μs and an intensity of 30–50 μA. At the onset of stimulation, intensity settings were increased until orofacial or contralateral forepaw dyskinesias were observed in order to confirm stimulation delivery, and immediately following a positive dyskinetic response, the intensity was set below the lower limit of dyskinesias, such that no rat was functionally impaired by stimulation. Rats assigned to INACTIVE stimulation did not receive stimulation at any time but were physically connected within their stimulator bowls to a commutator. All rats were monitored carefully and frequently (at least twice and often four to five times per day) over the course of the study with special regard for maintaining the connection between the electrode lead and the commutator and for stimulus isolator battery levels. Stimulus isolator batteries generally last for several days and up to one week. Stimulus isolators with low battery power make a warning sound that would prompt switching the isolator out for a newly charged unit.

### Sacrifice

After the stimulation interval, rats were deeply anesthetized (60 mg/kg, pentobarbital, i.p.) and perfused intracardially with heparinized saline at 37°C followed by ice-cold, 4% paraformaldehyde or ice-cold saline for lesioned and intact brains, respectively. Care was taken to minimize the tissue damage resulting from removing the skull with the electrode still intact. Lesioned brains were post-fixed in 4% paraformaldehyde for 24 hours and transferred to 30% sucrose in 0.1 M phosphate buffer. Unlesioned, saline-perfused brains were immediately flash-frozen in 3-methyl butane and stored at -80°C.

### Microdissections

Brains acclimated to -20°C for at least one hour and then sectioned into 1–2 mm slabs using chilled, single-edge razor blades and a chilled, aluminum brain block. The hippocampus, M1 cortex, SN, striatum and the ventrolateral/ventroanterior thalamus were dissected on a cold plate set at −12°C (ThermoElectric Cooling America Corp, Chicago, IL) using a chilled, small tissue punch. Slabs containing the EP were examined for visual verification of electrode placement prior to dissection of this nucleus. Hippocampal and thalamic tissue punches of 2.0 mm in diameter were taken from a 1.0 mm-thick slab immediately dorsal to the STN (between AP -3.5 and AP -4.5). Hippocampal punches contained all three CA subdivisions; thalamic punches contained posterior, ventral posterior medial, ventral posterior lateral and lateral posterior nuclei. Each structure was placed in a pre-chilled microcentrifuge tube and stored at -80°C.

### Protein assay

Total protein levels were quantified by comparison to a bicinchoninic acid (BCA) standard curve. Tissue was first homogenized in T-PER (Pierce, Rockford, IL) using a 300 V/T Ultrasonic Homogenizer (BioLogics, Manassas, VA). From each sample, 20 μl was removed, added to 20 μl of 2% SDS solution and then added to a BD Falcon 96-well Microtest plate (Fisher, Morris Plains, NJ) along with a BSA standard curve (Pierce, Rockford, IL). CuSO_4_ (4%) was added to each well, and the plate was incubated at 37°C for twenty minutes. Each plate was read at 590 nm on a spectrophotometer (Thermo Scientific).

### BDNF ELISA

An ELISA for BDNF was performed in triplicate in Nunc microwell 96-well microplates (Thermo Scientific) as per the manufacturer’s instructions (BDNF Emax ImmunoAssay Systems Kit, Promega, Madison, WI). Each plate was read at 450 nm on a spectrophotometer (Thermo Scientific), and unknown values were calculated via interpolation against a BDNF standard curve. Each structure was analyzed individually with ACTIVE and INACTIVE groups present on each plate. Results were calculated as BDNF pg/mg of total protein. The data were normalized relative to the average value of the structure on the intact side (i.e., contralateral to the electrode) of the INACTIVE group within the same plate and then averaged across plates. Normalization was calculated such that the potential effects of electrode implantation could be differentiated from the effects of stimulation.

### Tyrosine hydroxylase immunohistochemistry for SNpc neurons

Paraformaldehyde-perfused and postfixed brains were frozen on dry ice and sectioned at 40 μm thickness using a sliding microtome in six series. One series (i.e., every sixth section) was stained with antisera for tyrosine hydroxylase (TH) using the free-floating method. Tissue was blocked in serum and incubated overnight in primary antisera directed against TH (Chemicon MAB318, mouse anti-TH 1:4000). Cell membranes were permeabilized with the addition of Triton-X (0.3%) to the 0.1 M Tris buffer during incubations. Sections were then incubated in biotinylated secondary antisera against mouse IgG (Chemicon AP124B, 1:400) and followed by the Vector ABC detection kit employing horseradish peroxidase (Vector Laboratories, Burlingame, CA). TH immunoreactive (THir) neurons were visualized upon exposure to 0.5 mg/ml 3,3’-diaminobenzidine (DAB) and 0.03% H_2_O_2_ in Tris buffer. Sections were mounted on subbed slides, dehydrated with ethanol and then xylenes and coverslipped with Cytoseal (Richard-Allan Scientific, Waltham, MA).

### Kluver-Barrera histology

**Fig 2:**
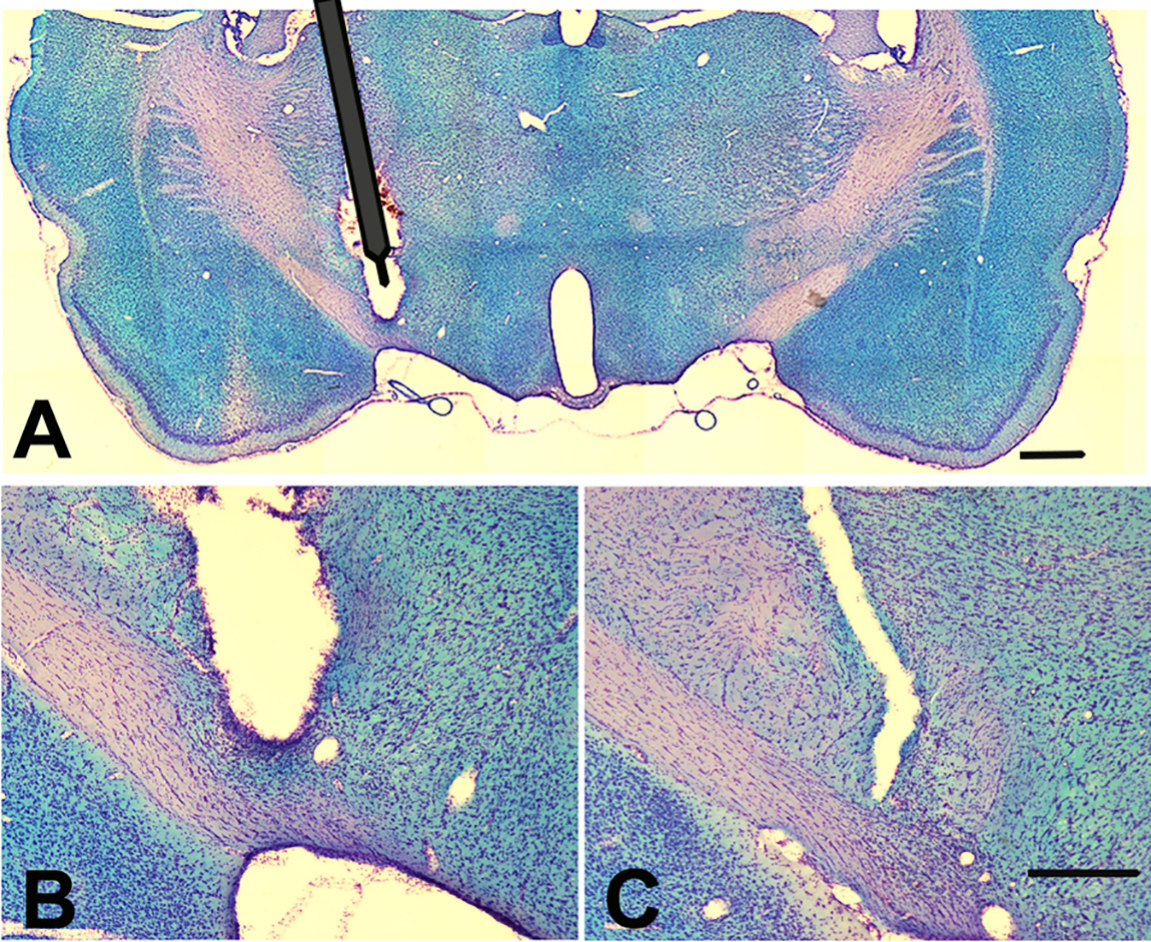
Electrodes implanted in the EP remain in position over the two-week stimulation interval. Representative photomicrographs illustrate unilateral electrode placement in the EP following Kluver-Barrera staining. (**A**) Low magnification image shows the approximate placement of the stimulating electrode prior to its removal post mortem and the tissue damage related to the removal process. The active electrode tip diameter is 150 μm whereas the shaft of the electrode is 400 μm in diameter. (**B**) High magnification of the electrode tip’s position in the EP. **(C)** EP neurons are visible in a nearby coronal section (≈160 μm caudal), indicating that a significant portion of the EP remained intact. Rats in which electrodes were found to be positioned more than 250 μm away from the EP were excluded from analysis based on previous estimates of current spread [20]. Scale bar in A = 1000 μm, C = 500 μm.

Every sixth section of the entopeduncular nucleus (EP) was stained using Kluver-Barrera histochemistry [24] to evaluate for appropriate targeting of the electrode to the EP. Only rats with correctly positioned electrodes were included in the data analysis for Experiment 2. Electrode location was considered to be appropriate if the tip of the electrode was observed within 250 μm of the border of the EP within any of the sections based on previous studies in which current spread was determined [19]. A representative histological section is shown in Fig 2.

### Unbiased Stereology of THir Neurons in the SNpc

The number of THir neurons in the SNpc ipsilateral and contralateral to 6-OHDA injection was quantified using unbiased stereology with the optical fractionator principle. Using a Nikon Eclipse 80i microscope, Retiga 4000R (QImaging, Surrey, BC, Canada) and Microbrightfield StereoInvestigator software (Microbrightfield Bioscience, Burlingame, VT), THir neuron quantification was completed by drawing a contour around the SNpc borders at 4X, and THir neurons were counted according to stereological principles at 60X (NA 1.4), providing more than sufficient resolution for precise stereology. As previously described [25], a 50 x 50 μm counting frame was used on a 273 x 119 μm grid size, and the tissue thickness was optically measured at every site with a top guard zone height of 5.0 μm used. THir neurons were counted if the top focal plane of the soma was identified within the dissector. Percent remaining THir neurons of the ipsilateral, lesioned SNpc relative to the contralateral, intact SNpc were also calculated. The Schmitz-Hof Coefficients of Error were less than or equal to 0.15 for all analyses.

### Selective Total Enumeration of THir Neurons in the SNpc

The number of THir neurons in the SNpc ipsilateral and contralateral to 6-OHDA injection was also quantified using selective total enumeration, a modified stereological method previously established to accurately quantify nigral THir neurons following intrastriatal 6-OHDA injected using identical parameters [25]. SNpc THir neurons from three sections, easily identified by the presence of the medial terminal nucleus of the accessory optic tract (AP -5.04 mm, ML -5.28 mm, DV -5.52 mm relative to bregma [26]) were quantified. Using a Nikon Eclipse 80i microscope, Retiga 4000R (QImaging, Surrey, BC, Canada) and Microbrightfield StereoInvestigator software (Microbrightfield Bioscience, Burlingame, VT), selective total enumeration THir neuron quantification was completed by drawing a contour around the SNpc borders at 4X. Virtual markers were then placed on THir neurons at 20X and quantified. Total THir neuron numbers in the intact or lesioned SNpc were summed for the three MTN sections counted. Percent remaining THir neurons of the ipsilateral, lesioned SNpc relative to the contralateral, intact SNpc were calculated, as reporting of raw data is inappropriate for what should only be considered a relative metric of the SN from one hemisphere versus the other side.

### Statistical Analysis

All statistical analyses were performed using IBM SPSS Statistics (IBM, Armonk, NY). BDNF expression levels in ‘Experiment 1’ were confirmed by a repeated-measure (RM)-ANOVA. Values presented are normalized to expression levels on the side contralateral to the electrode ± SEM. In ‘Experiment 2’, a two-way RM-ANOVA followed by a least significant difference *post hoc* analysis was conducted to confirm the presence of functional deficits and the behavioral response to DBS. Differences in THir neuron survival were determined by a two-tailed Student’s t-test, either comparing the unlesioned hemisphere to the lesioned hemisphere or comparing lesioned hemispheres between ACTIVE and INACTIVE groups. Statistical significance was set at *p* < 0.05. Statistical analyses are summarized in Table 1.

**Table 1:** Statistical Table.

	Data Structure	Type of Test	Power
a	Normal Distribution	RM-ANOVA, pairwise comparison	0.68
b	Normal Distribution	RM-ANOVA, pairwise comparison	0.998
c	Normal Distribution	RM-ANOVA, between subjects comparison	0.063
d	Normal Distribution	RM-ANOVA, within subjects comparison	0.574
e	Normal Distribution	RM-ANOVA, pairwise comparison	N/A
f	Normal Distribution	RM-ANOVA, pairwise comparison	N/A
g	Normal Distribution	RM-ANOVA, pairwise comparison	N/A
h	Normal Distribution	RM-ANOVA, pairwise comparison	N/A
i	Normal Distribution	Student's T-Test	1.0
j	Normal Distribution	Student's T-Test	0.57
k	Normal Distribution	Split-Plot ANOVA	0.507
l	Normal Distribution	Split-Plot ANOVA	0.05
m	Normal Distribution	Split-Plot ANOVA	0.095
n	Normal Distribution	Split-Plot ANOVA	0.050
o	Normal Distribution	Split-Plot ANOVA	0.100

## Results

### Qualitative Examination of the EP

The EP spans approximately 900 μm along the rostral-caudal axis. In our one in six, 40 μm series used for Kluver-Barrera the EP is normally present in four sections. In all cases, rats were only included in the study if the EP was visible in at least three sections and if the electrode was located at the dorsal border of the EP, demonstrating no lesion of the nucleus. Representative sections are shown in Fig 2.

### Short-Term Behavioral Response to DBS of the EP

Rats receiving ACTIVE stimulation were evaluated for behavioral responses to EP stimulation for a thirty-minute interval upon initiation of stimulation. Stimulation was slowly increased until the onset of dyskinesias or rotations. Contralateral orofacial dyskinesias often appeared first with stimulation amplitudes between 30–90 μA, though most appeared under 50 μA. By increasing current amplitude, rotations to the ipsilateral side and dyskinesias of the contralateral forepaw were elicited, usually within 20 μA of the amplitude of orofacial dyskinesias, the vast majority observed below 60 μA. These dyskinesias persisted over the initial thirty-minute interval; hence, current intensity was lowered to below the threshold of dyskinesias for long-term stimulation. A similar profiling of behaviors elicited by stimulation has been reported previously [20, 21, 27].

### EP stimulation does not improve forelimb asymmetry

**Fig 3:**
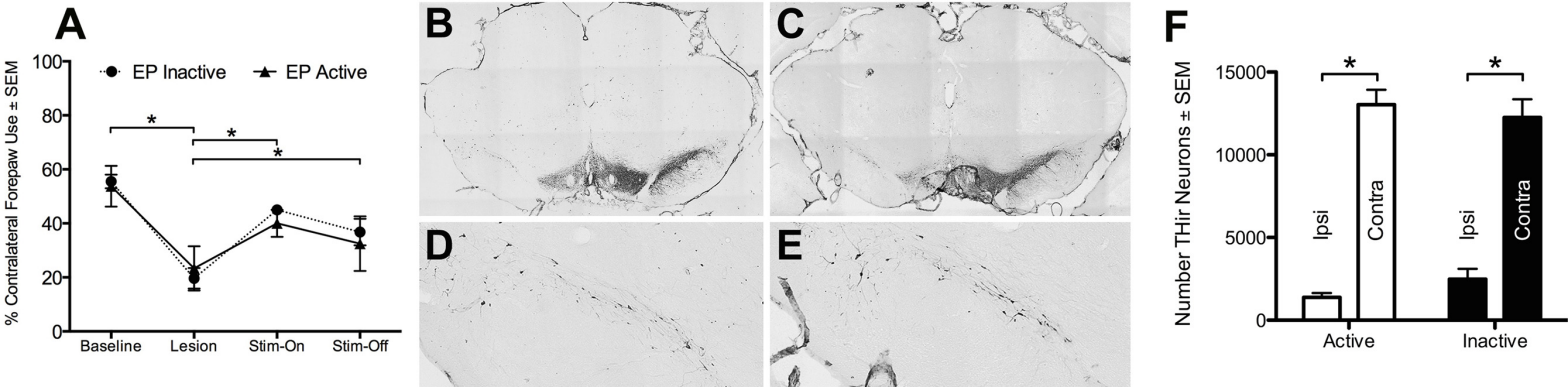
EP DBS does not correct forelimb asymmetry or provide neuroprotection from 6-OHDA. **(A)** Rats receiving intrastriatal 6-OHDA followed by either ACTIVE or INACTIVE EP DBS were analyzed for forelimb use asymmetry in the cylinder. 6-OHDA led to a significant decrease in contralateral forelimb use. However, ACTIVE EP DBS showed no difference compared to INACTIVE DBS at any time point. Of note, two weeks following electrode implantation in the EP, an improvement in contralateral forepaw use was observed. **(B-E)** EP DBS does not provide neuroprotection from 6-OHDA. Neither ACTIVE nor INACTIVE EP DBS halted ongoing nigral DA neuron loss normally observed between two and four weeks after intrastriatal 6-OHDA. (**B-C)** Representative nigral sections from both INACTIVE (**B**) and ACTIVE (**C**) EP DBS rats labeled with TH antisera reveal significant depletion of nigral DA neurons in the lesioned hemisphere. **(D-E)** At higher magnification, DA neuron loss appears equivalent between the INACTIVE (**D**) and ACTIVE (**E**) treatment groups. (**F**) Stereological assessment of THir neurons revealed a significant effect of 6-OHDA administration but no significant difference between ACTIVE and INACTIVE EP DBS groups.

Two weeks following intrastriatal 6-OHDA, a significant decrease in contralateral forelimb use was observed in both ACTIVE and INACTIVE groups (*F*_(3,6)_ = 5.403, *p* = 0.038^a^ and *F*_(1.718,18)_ = 21.137, *p* < 0.001^b^, respectively). Specifically, rats lesioned with 6-OHDA reduced contralateral forelimb use compared to baseline by over half as much. A two-way RM-ANOVA revealed no significant difference between treatment groups (*F*_(1,8)_ = 0.147, *p* = 0.712^c^) but did reveal a significant main effect within subjects (*F*_(3,24)_ = 20.335, *p* < 0.001^d^); therefore, the ACTIVE and INACTIVE treatment groups were combined for pairwise comparisons within subjects. Intrastriatal 6-OHDA resulted in deficits in contralateral forelimb use compared to baseline (*p* < 0.001^e^) that persisted for the duration of the study (*p* = 0.007^f^). However, contralateral forelimb use was significantly improved (compared to the two-week, post-lesion time point) at both four-week time points regardless of whether stimulation was ‘on’ or ‘off’ (*p* = 0.001^g^ and p = 0.015^h^, respectively). These results demonstrate no functional impact of ACTIVE stimulation on contralateral forelimb use but a significant improvement over time in both treatment groups. These results are depicted in Fig 3A.

### EP Stimulation Does Not Provide Neuroprotection for SNpc Neurons

Rats in the intrastriatal 6-OHDA, INACTIVE treatment group possessed significantly fewer SNpc THir neurons ipsilateral to the injection compared to the contralateral SNpc (*t*_(20)_ = -12.117, *p* < 0.001^i^). Specifically, the unlesioned SNpc in INACTIVE rats possessed 12255 ± 1099 THir neurons whereas the lesioned SNpc contained 2482 ± 619, or ≈83% fewer THir neurons than the unlesioned SNpc, as expected from this lesion paradigm [20, 25]. Similarly, rats that received two weeks of continuous EP stimulation also displayed a significant depletion of ≈90% THir neurons ipsilateral to 6-OHDA (ACTIVE unlesioned = 13029 ± 897; ACTIVE lesioned = 1379 ± 268). No significant difference was observed in the magnitude of degeneration measured in ACTIVE vs. INACTIVE rats (*t*_(7.193)_ = 2.136, *p* = 0.069^j^). These results are illustrated in Fig 3B–3F.

Selective total enumeration of THir SNpc neurons [25] was also used to assess lesion status and to compare its utility versus unbiased stereology for our laboratory’s future use. Raw counts from the three sections where the MTN fibers are present are as follows: Inactive rats had 593 ± 34 and 139 ± 25 SNpc neurons on the unlesioned and lesioned sides, respectively; Active rats had 648 ± 40 and 105 ± 9 SNpc neurons on the unlesioned and lesioned sides, respectively. As previously reported, direct comparisons of estimates of lesion severity (and SEM) determined using selective total enumeration closely approximated those determined using unbiased stereology: ≈75% (±5.2) and ≈85% (±1.2) fewer THir neurons than the unlesioned SNpc in INACTIVE and ACTIVE rats, respectively.

### Impact of EP DBS on BDNF Protein Levels

**Fig 4:**
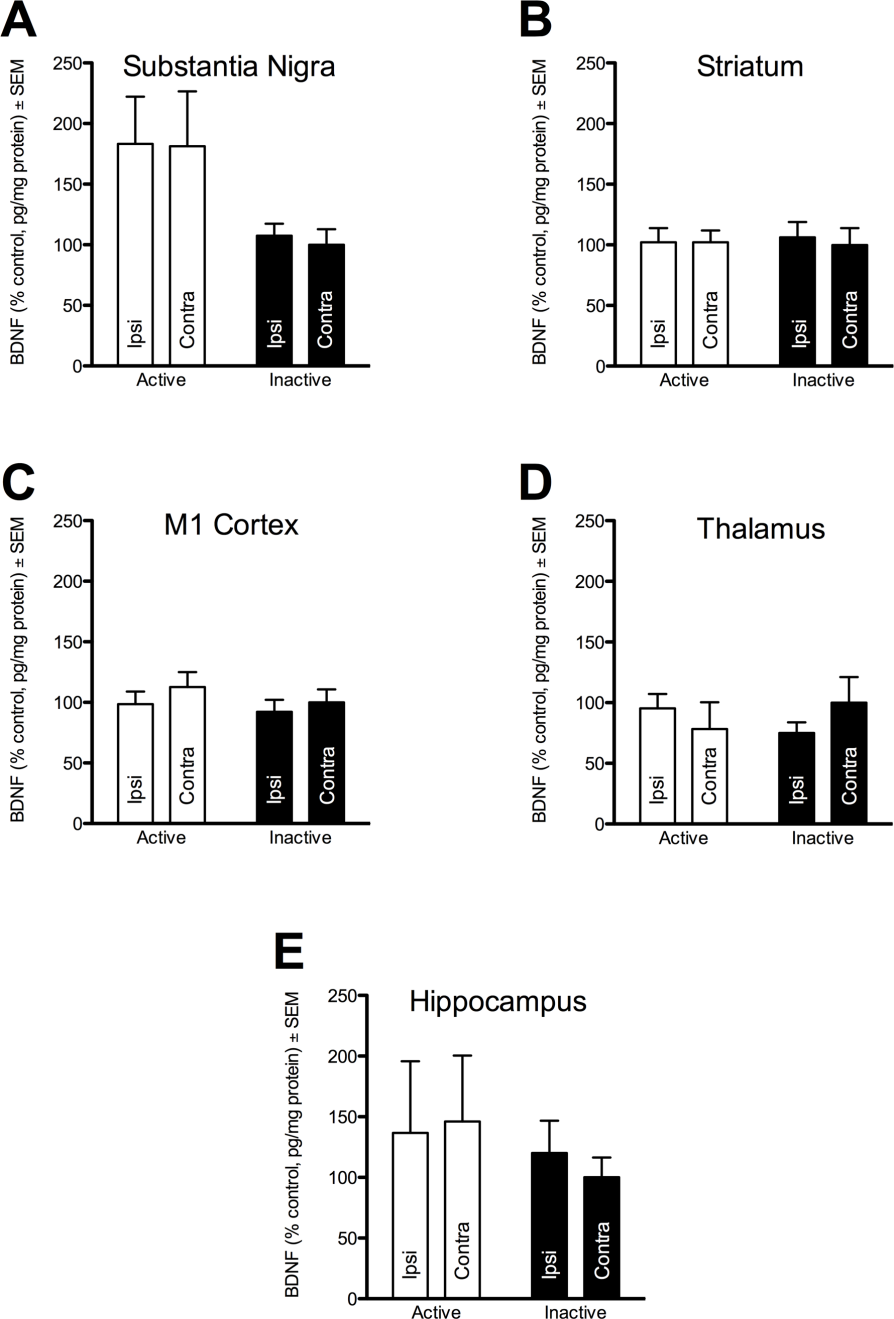
EP DBS does not increase BDNF. BDNF protein levels were normalized to total protein in key basal ganglia structures of intact rats after a two-week stimulation interval. Data from each structure were normalized to the corresponding structure from the INACTIVE, contralateral (to 6-OHDA and electrode lead) hemisphere to control for the potential effect of dopamine denervation or electrode implantation on BDNF levels. Samples were obtained for the ipsilateral (Ipsi) and contralateral (Contra) substantia nigra (SN), striatum (STR), primary motor cortex (M1), thalamus and hippocampus. No significant difference was observed between ACTIVE and INACTIVE stimulation groups nor within animals between sides, though there was a trend toward significance between the Active and Inactive SN bilaterally.

Five structures—namely, the SN, striatum, M1 cortex, thalamus and hippocampus—were examined bilaterally for levels of BDNF protein expression in unlesioned rats that received unilateral EP stimulation for two weeks (ACTIVE) or INACTIVE electrode-implanted controls. ACTIVE stimulation of the rat EP was not associated with a significant increase in BDNF protein levels in any of the structures examined (viz., SN, thalamus, hippocampus, striatum and M1; *p* > 0.05^k-o, respectively^), although BDNF levels in the SN of ACTIVE rats displayed a trend toward increased BDNF (*F*_(1,16)_ = 4.426, *p* = 0.052^k^). BDNF protein levels were not measured for the EP since they are below the detection threshold for an ELISA [21]. Of note, our raw data are generally of a similar magnitude as our previous work [21] and others [28], suggesting consistency with the assay. The comprehensive results are summarized in Table 2 and Fig 4.

**Table 2:** Measured BDNF Levels by Structure.

Structure	BDNF (pg/mg) ± SEM			
	ACTIVE		INACTIVE	
	Ipsilateral	Contralateral	Ipsilateral	Contralateral
**Substantia Nigra**	32.86 ± 5.71	33.53 ± 7.70	18.70 ± 2.05	17.45 ± 2.47
**Striatum**	7.41 ± 1.50	8.13 ± 2.20	6.67 ± 2.73	5.37 ± 1.79
**M1 Cortex**	11.49 ± 1.83	12.79 ± 1.66	9.48 ± 1.40	10.04 ± 1.14
**Thalamus**	5.23 ± 0.65	4.30 ± 1.21	4.12 ± 0.47	5.49 ± 1.16
**Hippocampus**	24.03 ± 10.38	25.68 ± 9.55	21.09 ± 4.68	17.58 ± 2.86

## Discussion

In the present study, we investigated whether long-term EP DBS provides neuroprotection similar to the neuroprotection observed following long-term STN DBS employed in the same lesion paradigm [20]. EP DBS was not associated with stimulation-dependent, functional improvements in contralateral forelimb use. Further, long-term EP DBS during the degenerative process initiated by 6-OHDA did not provide neuroprotection of nigral DA neurons or significant changes in BDNF levels within the brain. These results are in stark contrast with the effects of STN DBS in rodent and non-human primate models. As previously reported by several laboratories [21, 27, 29–38], STN DBS in rats is associated with functional improvement in amphetamine-induced contralateral rotations, treadmill locomotion, walking speed, forelimb akinesia, rearing activity and reaction time following intrastriatal 6-OHDA. In contrast, our present study fails to demonstrate stimulation elicited functional improvement in the cylinder task; admittedly, we did not employ other behavioral tasks due to constraints with chronic stimulation using our DBS platform. The improvement we observed in forelimb use was independent of stimulation status and only observed four weeks post surgery, suggesting that plasticity following the partial lesion of the EP may be responsible for the observed motor improvements, as has been reported previously for lesions of the EP [39] and for microlesions of the thalamus [40, 41]. Of note, under identical 6-OHDA lesion parameters, functional improvement in contralateral forelimb use does not occur spontaneously or in association with implantation of INACTIVE electrodes in the STN [21, 25], nor is the STN microlesioned by implantation of the same electrode used in this study [20]. Therefore, it appears that the functional improvement we observe in the present study is specific to implantation of an electrode in the EP.

Whereas there have been numerous reports of STN DBS-mediated functional improvements in rats [21, 27, 29–38], only two reports by Summerson and colleagues demonstrate motor effects following EP DBS [42, 43]. Specifically, amphetamine-induced rotations were acutely attenuated by stimulation amplitudes higher than used in the present study (viz., 65–100 μA [42]). Curiously, although forelimb akinesia was used in the first Summerson report to screen for 6-OHDA-induced dopamine depletion, the cylinder task was not used to assess the functional impact of EP DBS [42]. In their second report, Summerson and colleagues show behavioral improvement in the cylinder task, but again, under conditions of high current amplitudes [43]. In our present study, as reported previously [20, 27], amplitudes above 50 μA elicited contralateral dyskinetic movements, beginning with the orofacial area and forepaw. With even higher stimulation intensity levels, we also observed the tendency to rotate in the contralateral direction. Given that increases in current lead to an expanding volume of tissue activation (termed in [44]), we would expect that at higher amplitudes neurons/circuits outside of the EP would be recruited, as has been previously determined with rat STN stimulation [19]. Modulation of circuitry outside the EP may be responsible for the limited, observed functional effects reported with EP stimulation at higher stimulation amplitudes [42, 43, 45]; indeed, rotational responses have been associated with direct SN stimulation [46, 47]. Although it is possible that we did not use the precise combination of stimulation intensity, pulse width and frequency required to elicit functional improvements, we can confirm that the current of 65–100 μA reported by Summerson and colleagues would have been incompatible with both long-term stimulation as well as accurate forelimb usage. We speculate that non-specific stimulation outside of the EP may be involved in these previously observed functional effects.

In the present study we chose to target the rat EP due to its perceived homology to the primate GPi. Whereas remarkable similarities exist between the rat EP and the primate GPi in both the γ-aminobutyric acid (GABA) phenotype and afferent/efferent connectivity, an important difference in their firing properties remains (Table 3). The primate GPi is composed of fast-spiking, pacemaking neurons, whereas the rat EP is not [48–50]. In this regard the rat SN pars reticulata (SNpr) is more similar to the primate GPi (Table 3); indeed, stimulation of the SNpr in rats has been shown to improvement forelimb akinesia [51]. The disparity in firing properties between the rat EP and primate GPi may underlie the inability of focused EP DBS to ameliorate functional deficits induced by dopamine denervation. Our results suggest that stimulation of the rat EP may not serve as an appropriate model for GPi DBS for PD.

**Table 3:** Comparison of Primate GPi, Rat EP, Primate SNpr and Rat SNpr.

Feature	Primate GPi	Rat EP	Primate SNpr	Rat SNpr	References
GABAergic	Yes	Yes	Yes	Yes	[69–73]
Major Basal Ganglia Output	Yes	Yes	Yes	Yes	[64, 65, 71]
Afferents from STN, Striatum & GPe	Yes	Yes	Yes	Yes	[55–57, 71, 74–81]
Efferents to Thalamus & PPN	Yes	Yes	Yes	Yes	[65, 66, 82–87]
Efferents to Lateral Habenula	Yes	Yes	No	No	[64, 65, 88, 89]
Fast-Spiking Pacemaker	Yes	No	Yes	Yes	[50, 51, 54, 71, 90]

GPi = globus pallidus interna, EP = entopeduncular nucleus, SNpr = substantia nigra pars reticulata, GABA = γ-aminobutyric acid, STN = subthalamic nucleus, GPe = globus pallidus externa, PPN = pedunculopontine tegmental nucleus

Several laboratories have shown that long-term STN DBS in rats and non-human primates is associated with neuroprotection of nigral DA neurons from 6-OHDA- or 1-methyl-4-phenyl-1,2,3,6-tetrahydropyridine (MPTP)-induced degeneration, respectively [20, 27, 52–54]. It also has been demonstrated that high-frequency stimulation of the STN provides a significant level of neuroprotection and essentially halts the nigral degeneration that normally manifests during that period [20]. In contrast, continuous EP stimulation over the identical post-6-OHDA interval does not protect SNpc DA neurons from the continuing nigral degeneration induced by 6-OHDA. Both ACTIVE and INACTIVE EP DBS treatment groups displayed equal levels of nigral neuron loss commensurate with the normal lesion magnitude over this time course [20].

While the mechanism of STN DBS-mediated neuroprotection in the 6-OHDA PD rat has not been elucidated, the disparity in protection of SN neurons between STN and EP stimulation may involve BDNF. In unlesioned rats STN DBS is associated with a significant, threefold increase in BDNF in the striatum and a non-significant increase in the SN [21]. Whereas a similar non-significant increase in BDNF in the SN is observed with EP DBS, striatal BDNF levels were not affected. We hypothesize that these contrasting results are due to differences in the phenotype and neural circuitry associated with the stimulation sites. The STN sends glutamatergic projections to the SNpc, the SNr and the striatum [55–58]. Stimulation of glutamatergic, hippocampal neurons *in vitro* results in activity-dependent BDNF release [59, 60]. Ergo, high-frequency stimulation of the glutamatergic neurons of the STN, known to contain BDNF [61, 62], may similarly mediate an increase in BDNF in STN target sites and provide trophic support for nigral neurons [63]. The EP sends GABAergic projections to the pedunculopontine nucleus (PPN), the lateral habenula, the centromedian nucleus and the ventral anterior and the anterior part of the ventral lateral thalamic nuclei [64–66] (Table 3). Although we did not measure BDNF levels in the PPN in the present study, we show that EP DBS does not significantly increase BDNF in either the striatum or the SN. Further, while it is well established that glutamatergic neurons secrete BDNF [67], it is unclear whether GABAergic EP neurons possess or release BDNF. Analysis of BDNF expression levels in the Allen mouse brain atlas (2012) indicate expression of BDNF in the STN but levels indistinguishable from background in the EP. Other studies have demonstrated a differential effect on BDNF signaling within the hippocampus following STN or EP stimulation [21, 45]. Of note, our non-significant increase in SN BDNF in the ACTIVE group may be explained by current spread from the EP to the STN, thereby replicating our previous work [68]; the high variance in these data despite a similar group size to our previous work [68] is consistent with this explanation. Further studies are required to elucidate the source of elevated nigrostriatal BDNF following STN DBS and whether this phenomenon contributes to the neuroprotection observed.

## Conclusions

In summary, EP DBS in our rat model of PD does not result in functional improvements nor morphological neuroprotection. Further, EP DBS in the rodent does not result in significant increases in BDNF protein levels in the nigrostriatal system or M1 cortex. Studies directly comparing STN vs. GPi stimulation in the non-human primate are warranted to ascertain whether our rat findings are due to the neuroanatomical differences between the rodent EP and the primate GPi. STN DBS has been shown to be neuroprotective in the non-human primate [53]. If GPi stimulation fails to provide neuroprotection in the non-human primate, this would suggest that stimulation of the STN but not the GPi may offer a disease-modifying effect in PD patients.

## Supporting Information

S1 File Raw Data. An Excel-based spreadsheet containing all of the raw data analyzed, including percentages of contralateral forelimb use from the cylinder task, stereological quantification of THir neurons of the SN, total enumeration of THir SNpc neurons from sections containing MTN fibers and data in pg/mg of protein from BDNF ELISA.
(XLSX)
